# Ensuring Knowledge Transfer: The Critical Role of “What-If” Discussions in Virtual Patient Simulation

**DOI:** 10.5334/pme.2363

**Published:** 2026-08-05

**Authors:** Zhien Li, Maryam Asoodar, Tom Keulers, Desirée Joosten-ten Brinke, Diana Dolmans

**Affiliations:** 1 Department of Educational Development & Research, School of Health Professions Education, Faculty of Health, Medicine and Life Sciences, Maastricht University, Maastricht, The Netherlands; 2 Department of Radiation Oncology (Maastro), GROW School for Oncology and Reproduction, Maastricht University Medical Centre, Maastricht, The Netherlands

## Abstract

**Background:**

Virtual Patient (VP) systems are widely used in medical education, yet evidence on how they support near and far transfer remains limited. This study investigates integrating VPs with role modelling, varied case practices, and “what-if” discussions. It aims to measure the students’ overall experience with the intervention, improvement in performance scores on near- and far-transfer cases, and to explore how and why the intervention is perceived to enhance transfer.

**Methods:**

An exploratory mixed-methods study was conducted with 21 second-year undergraduate medical students enrolled in the course “Personalised Medicine in Cancer Treatment and Care”. Students engaged in role modelling performed by an oncologist, worked through authentic cases with varying patient characteristics, and participated in structured “what-if” discussions. Perceptions about the overall experience were gathered through a 19-item survey (n = 21). Performance scores were evaluated using pre- and post-assessments involving VPs and paper-based cases designed to measure near transfer (Case D) and far transfer (Case E) (n = 21). Group interviews (n = 8) provided deeper insights into enhancing transfer.

**Results:**

Survey results revealed positive student perceptions of role modelling (M = 4.44), varied case practices (M = 4.35), and “what-if” discussions (M = 3.76). Significant improvements were observed in tumour-stage reasoning for both near and far transfer (p < .001), in initial patient impressions (p = .007) for far transfer, and in the total score (p = .018) for far transfer. Thematic analysis indicated that students benefited from a stepwise build-up in case complexity, recognition of similar reasoning steps across cases, reflective “what-if” discussions that highlighted underlying principles, and peer dialogue around VP feedback that supported concept abstraction.

**Conclusions:**

Students evaluated the VP session positively and performance data indicated significant improvements, suggesting that the intervention enhanced near and far transfer. Reflection through “what-if” discussions was perceived to deepen understanding of the underlying principles enabling transfer.

## Introduction

In education, students are confronted with cases based on real-life or authentic professional tasks, for instance, varied workplace situations, because this type of situation helps them to integrate knowledge, skills and attitudes and adequately prepares them for practice [1]. Virtual Patients (VPs) provide good opportunities to practice different scenarios in a safe environment [2,3]. To enhance transfer to practice, the VP cases that are offered to students should differ from each other in an authentic context, because this “variability of practice” will help transfer of learning [4,5]. Transfer of learning is essential in medical education because clinical reasoning skills developed in one case must be flexibly applied to patients with varying characteristics and contexts [6]. For example, the same disease may require different diagnostic or treatment decisions depending on the patient’s age, comorbidities, or lifestyle factors [7,8].

According to Perkins and Salomon’s definition, transfer of learning refers to the ability to apply prior learning in new situations [7]. It involves near transfer, where knowledge is applied in contexts that are quite similar to the original learning situation, and far transfer, where more abstract principles are applied in novel or structurally different contexts [7]. Literature suggests that near and far transfer can both be facilitated through practice with a variety of cases and by providing scaffolding [4,9]. Both near and far transfer are enhanced by stimulating abstraction and analogical reasoning across cases [9,10].

The transfer process is supported by abstraction, where learners generalize underlying principles beyond surface features, and by recognizing structural similarities across contexts, which allow them to apply prior learning to new contexts [11,12,13,14,15]. In this study, near transfer refers to applying knowledge learned from one neck cancer case to another case within the same domain. Far transfer refers to applying the same underlying reasoning principles to a different domain, for example, from neck cancer case to lung cancer case.

Building on theoretical perspectives, an important question concerns how variability in case design is implemented within VP learning environments to enhance knowledge transfer. VPs offer practical opportunities by engaging learners in realistic, case-based reasoning across different patient contexts. Research [16] has found that medical students perceived VPs as a valuable resource for near transfer to a similar case and far transfer to novel patient settings. While VPs offer clear potential for promoting transfer, the growing move toward embedding them in structured learning designs calls for further work to understand how such integration supports students’ abilities to apply knowledge across similar and novel contexts [17,18].

Dubé and Ducharme [19] found that, in nursing training, “what-if” discussions not only enhance practical skills but also facilitate the transfer of learning. “What-if” reasoning is a pedagogical approach that allows students to consider alternative outcomes based on different clinical decisions driven by varied clinical situations [20]. Subsequent studies indicate that “what-if” questions encourage reflective reasoning that helps learners link specific clinical decisions to the underlying principles governing different patient scenarios [21,22].

While previous studies have acknowledged the value of reflective “what-if” discussions in supporting transfer [19,21], these studies primarily relied on self-reported confidence rather than observed performance on novel cases. Moreover, most studies typically refer to “transfer” in a broad sense without distinguishing whether the learning was applied to “quite” similar situations (near transfer) or to novel, structurally different situations (far transfer), leaving open the question of whether “what-if” discussions can measurably enhance both types of transfer.

We aim to bridge this gap by examining whether integrating VPs with role modelling, practice on varied authentic cases, and structured “what-if” discussions can facilitate medical students’ clinical reasoning and support both near and far transfer.

Research Questions:

RQ1: What are students’ overall perceptions of integrating VPs with role modelling, varied case practice, and “what-if” discussions?

RQ2: Do VPs with role modelling, varied case practice, and “what-if” discussions improve students’ performance in near transfer and far transfer?

RQ3: How and why do VPs with role modelling, varied case practice, and “what-if” discussions enhance transfer to similar or novel cases?

## Method

### Setting and Participants

This exploratory study was conducted within the elective course “Personalised Medicine in Cancer Treatment and Care” at Maastricht University, offered to second-year medical students. The intervention examined in this study consisted of a single 110-minute session and the session was embedded within the regular course schedule rather than added as an extra activity. All 21 enrolled students participated in the VP session and consented to complete the survey. Eight students volunteered to participate in the group interviews held across three sessions. Participation in the group interviews was voluntary. Students were invited after the intervention session, and interview groups were formed based on availability.

### Intervention Design

**Figure 1:**
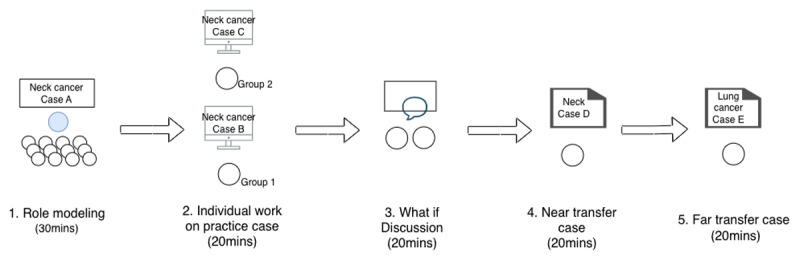
Study design.

To support the gradual development of clinical reasoning, the instructional approach for the VP cases followed a structured format specifically designed for this course (Figure 1). This design was informed by instructional principles suggesting that learning is enhanced when students first observe expert reasoning, then engage in varied practice, and are subsequently prompted to reflect on differences across cases to support abstraction and transfer of learning. Students first observed an expert role modelling demonstration with a neck cancer case (Case A), then practiced individually with varied VP cases (B or C). They subsequently engaged in structured “what-if” discussions to compare cases and encourage reflection, followed by individual paper-based tasks assessing near transfer (Case D) and far transfer (Case E).

1. *Role Modelling (30 min):* An expert demonstrated clinical reasoning using an authentic neck cancer case (Case A), providing students with a framework to guide their approach in subsequent cases and discussions.
2. *Individual Practice with Authentic Cases (20 min):* Subsequently, students were divided into two groups for **individual** VP practice. One group worked with VP Case B and the other with Case C, which differed in surface features such as patient age, comorbidities, and clinical presentation. The variations across cases are summarized in Appendix 1. Each case provided summative feedback designed by the expert from Case A, which served as input for the subsequent discussion. An example of the feedback interface provided to students is shown in Appendix 2.
3. *“What-if” Discussion (20 min):* After the VP exercises, students were paired with peers from the other group. They compared patient characteristics, reviewed the feedback, and addressed a set of structured “what-if” questions with a detailed discussion guide to explore how surface differences related to deeper clinical reasoning principles. For example, students reflected on questions such as “What if the patient were an 80-year-old female?” or “What if the patient had a history of diabetes?”. The full list of questions and the discussion guide are provided in Appendices 3 and 4. During the “what-if” discussions, faculty were present to monitor the session and answer procedural questions, but the case discussions themselves were primarily student-led.
4. *Near Transfer Case (20 min):* After students finished the “what-if” discussion, they were challenged to work individually on a near transfer neck cancer Case D on paper to apply their acquired knowledge and reasoning skills to a closed clinical scenario. This task consisted of multiple-choice questions and open-answer questions.
5. *Far Transfer Case (20 min):* Following the near transfer case, participants worked individually on another paper-based lung cancer case (Case E). While maintaining a similar structure to previous cases, this case required students to extend their reasoning to a novel clinical context.

### The Virtual Patient Cases

Five cases were developed to target key steps in clinical reasoning: a) initial evaluation of the patient presentation, b) selection of examinations with justification, c) diagnosis through accurate TNM (tumour-node-metastasis) staging and reasoning, and d) treatment plan tailored to patient characteristics and stage. Cases B and C were implemented on the P-Scribe platform (www.pscribe.nl), a web-based e-learning system based in the Netherlands, in which students worked through text-based clinical cases, made diagnostic and treatment decisions, and received expert-written summative feedback to stimulate subsequent discussion. Cases D and E were paper based and designed to assess near transfer in a comparable neck cancer scenario and far transfer in a novel lung cancer case (see Appendix 5). All cases were grounded in authentic cancer treatment but varied in patient characteristics such as age, comorbidities, and medical history.

### Instruments

*Survey*: A 19-item survey was developed by the research team (ZL, MA, and TK), drawing on theoretical principles of role modelling, varied case practice, and “what-if” discussion. To ensure content validity, the items were refined through collaborative discussions with the full authorial team, leveraging their collective expertise in oncology and medical education research. The survey assessed students’ perceptions across five main sections: overall experience, role modelling, varied case practice, “what-if” discussions, and knowledge transfer. The full list of survey items is provided in Appendix 6.

*Group Interview:* Semi-structured (group) interviews were conducted to gain a deeper understanding of how and why VPs with role modelling, varied case practice, and “what-if” discussions enhance near and far transfer. The interview explored how “what-if” questions supported reasoning and the application of knowledge to new cases. In addition, the interviews investigated how the role modelling and practice with authentic cases contributed to students’ understanding of the reasoning steps and their ability to approach subsequent cases. Sample questions included:

- In what ways did the “what-if” discussions enhance your understanding and reflection on the knowledge acquired?
- How did the “what-if” discussions prepare you to translate the learning from the neck cancer cases to the lung cancer case?

### Data Collection and Analysis

To answer the first research question, the survey data were analysed descriptively by calculating the mean and standard deviation across the overall experience and four scales (role modelling, varied cases, “what-if” discussions, and transfer).

The second research question was answered by comparing pre-test performance in VP Cases B and C with post-test performance in the near transfer case (D) and far transfer case (E). All cases required students to make clinical decisions and justify their reasoning. The practice results were assessed with a six-item rubric developed with two expert surgical oncologists (TK and FW), based on the course objectives (see Appendix 7). To ensure content validity, the experts iteratively refined the criteria to align precisely with the course objectives and the essential steps of clinical reasoning in oncology. The scoring was conducted by the main researcher and one of the oncology experts (ZL and TK). Based on the absolute-agreement definition [23], inter-rater reliability was high (ICC = 0.84, 95% CI [0.72, 0.92]). The remaining worksheets were scored by the first author.

Case D assessed students’ ability to apply knowledge to a comparable neck cancer scenario, whereas Case E evaluated transfer to a novel lung cancer context. Together, these tasks provided outcome measures for both near and far transfer.

Statistical analyses were conducted on performance data using the Wilcoxon signed-rank test, because the Shapiro–Wilk test indicated non-normality (*p* < .05). This non-parametric approach is appropriate for small, paired samples that do not meet normality assumptions. Pre-test (Cases B & C) scores were compared with post-test near (D) and far transfer (E) scores. Statistical significance was set at α = .05, following conventional thresholds used in educational research. In addition, effect sizes (r) were calculated to support interpretation of the magnitude of observed differences.

To answer the third research question, three group interviews (n = 8, group sizes = 2, 1, 5) were thematically analysed using a combined deductive–inductive approach. Following the six steps framework [24], we: [1] familiarized ourselves with the data, [2] generated initial codes, [3] searched for themes, [4] reviewed themes, [5] defined and named themes, and [6] produced the report.

The interviews were guided by theoretical principles including role modelling, the use of authentic cases, and “what-if” discussions. The group interviews were conducted by an external interview expert (WG) and one researcher (ZL). The group interviews were recorded, transcribed, coded, and ordered in initial themes by two members of the research team (ZL and MA). Both researchers have experience in medical education research and qualitative analysis. These themes were collaboratively reviewed and refined in discussions with all authors.

The quantitative and qualitative analyses were conducted within a concurrent mixed-methods design. The findings from both strands were subsequently compared and integrated through triangulation. In this process, themes identified in the group interviews were examined alongside the survey results to explore areas of convergence and divergence.

### Ethical approval

Ethical approval was granted by the Faculty of Health, Medicine and Life Sciences Research Ethics Committee, Maastricht University (FHML-REC/2024/059). Participants provided informed consent prior to participation and were informed that participation was voluntary, with the right to withdraw at any time without penalty. Data were collected and stored confidentially, used solely for research purposes, and no known risks were associated with participation.

## Results

### Survey Data

Students reported generally positive perceptions of the intervention across all scales (Table 1). The overall experience was rated highly (M = 4.48, SD = 0.51). The same holds for the other instructional components, role modelling (M = 4.44, SD = 0.39), practice with varied cases (M = 4.35, SD = 0.61) and perceived knowledge transfer (M = 4.33, SD = 0.70). Ratings for the “what-if” discussions were also positive (M = 3.76, SD = 0.81). Detailed item-level results are provided in Appendix 6.

**Table 1:** Descriptives of the survey scales on student perceptions of learning and knowledge transfer.


CATEGORY	ITEMS (n)	MEAN	SD	CRONBACH’S α

Overall experience	1	4.48	0.51	–

Role modelling	4	4.44	0.39	0.50

Practice with varied cases	4	4.35	0.61	0.57

“What-if” discussion	7	3.76	0.81	0.90

Knowledge transfer	3	4.33	0.70	0.74


Note. All items were rated on a 5-point Likert scale (1 = strongly disagree to 5 = strongly agree).

### Performance on paper-based exam cases

To evaluate knowledge transfer, pre-intervention scores (Cases B and C) were compared to post-intervention near transfer (Case D) and far transfer scores (Case E). Table 2 summarizes the median scores and Wilcoxon signed-rank test results for each rubric item.

**Table 2:** Learning outcome (n = 21).


QUESTION	MEDIAN (PRE-TEST - CASE B&C)	MEDIAN (POST-TEST NEAR - CASE D)	MEDIAN (POST-TEST FAR - CASE E)	WILCOXON STATISTIC (W) (NEAR TRANSFER)	p-VALUE (NEAR TRANSFER)	WILCOXON STATISTIC (W) (FAR TRANSFER)	p-VALUE (FAR TRANSFER)	EFFECT SIZE (NEAR TRANSFER)	EFFECT SIZE (FAR TRANSFER)

Q1 What is your first impression of the patient? (Max: 4)	3	3	4	29	0.12	8	0.007	0.39	0.73

Q2 Pick the examinations you need for diagnosing the patient (Max:4)	3.33	4	4	29	0.25	40.5	0.45	0.32	0.20

Q3 Please describe the rationale for each test (Max: 4)	3	3	3	22.5	0.10	33	0.21	0.45	0.33

Q4 What is the T, N, and M stage of this tumour? (Max: 4)	4	4	4	0	0.08	8.5	0.16	0.93	0.47

Q5 Please conclude the tumour stage and write down the reasoning behind it (Max: 4)	2	3	4	14	<0.001	12.5	<0.001	0.75	0.78

Q6 Which treatment should be treatment of the first choice, according to the patient’s characteristics and TNM-stage? (Max 4)	4	4	4	6	0.17	18	1	0.51	0

Total (Max 24)	19	20	21	77.0	0.19	41.5	0.018	0.30	0.53

Average (Max 4)	3.17	3.33	3.5						


For near transfer, most items showed no significant differences, except for tumour stage reasoning (Q5), which improved significantly. In contrast, for far transfer, students showed significant gains in both initial patient impressions (Q1) and tumour stage reasoning (Q5), while other components did not differ significantly.

When examining overall performance, no significant improvement was observed for the near transfer case (Median = 3.33; W = 77.0, p = 0.19, r = 0.30), whereas a significant increase was found for the far transfer case (Median = 3.50, W = 41.5, p = 0.018, r = 0.53).

### Group interview data

A total of eight students participated in three group interviews (groups of 2, 1, and 5). The interviews explored students’ perceptions of knowledge acquisition and transfer in clinical reasoning through the integration of VPs, role modelling, varied case practice, and “what-if” scenario discussions. Analysis revealed four key themes: “Step-by-step learning with increasing complexity facilitated learning”, “Recognizing the similar steps in clinical reasoning was helpful”, “Reflective thinking through “what-if” discussions led to deeper understanding of the underlying principles and enhanced knowledge transfer”, and “The peer dialogue on VP system feedback facilitated concept abstraction”.

#### Theme 1: Step-by-step learning with increasing complexity facilitated learning

Students mentioned that the sequential arrangement of learning activities facilitated a gradual learning process. They described how observing the expert demonstration first provided a structured understanding of clinical reasoning, which was then reinforced through subsequent case practice. As the tasks became more complex, students reported that they were able to build on their previous learning and increasingly apply prior knowledge to new cases, making the challenge of solving unfamiliar cases more manageable.

*We also learned to apply prior knowledge to this case. Think about the lungs and what kind of tissue it is and then how we can do imaging on that. (FG1, S2)*

*I think the most interesting part was that after seeing the first case, when I did the second one and the third one, I knew a lot more, and I could use the information I learned in the first and second one for the third case. So it builds up. (FG3, S1)*

#### Theme 2: Recognizing the similar steps between cases in clinical reasoning was helpful

Students noted that the cases shared a similar structure in terms of clinical reasoning. This consistency helped them understand how to systematically approach a patient case and apply the same reasoning process across different clinical situations. Through repeated exposure to cases with a similar structure, students reported becoming more familiar with the sequence of reasoning steps and the types of diagnostic decisions required. This familiarity helped them internalize the reasoning process and approach later tasks with greater confidence.

*First, we had an introduction by an expert. After that, we did the VP assignment, which followed a similar structure. It used a lot of the same steps. (FG2, S1)*

*Having the VP assignment first and then the paper case at the end helped because you already had an idea of what to expect. So, when it came to filling that one in, it felt easier and it wasn’t completely new anymore. (FG2, S1)*

#### Theme 3: Reflective thinking through “what-if” discussions led to deeper understanding of the underlying principles and enhanced knowledge transfer

Students reported that the “what-if” discussions encouraged them to reflect on their clinical decisions by comparing different patient scenarios. Through these discussions, they moved beyond procedural steps and considered the reasoning behind their decisions. By discussing similarities and differences between cases, students reported becoming more aware of the underlying principles guiding their clinical reasoning, which they perceived as helpful when approaching new cases.

*It was helpful to compare the two cases and realize that the treatment I chose for my patient didn’t fit my partner’s case because of key differences. It felt similar to a “what-if” exercise – thinking through how things would change in a different context. (FG2, S1)*

*When you really talk about the mistakes you made, and how the patients differed, that’s when you truly learn about clinical reasoning… You notice the similarities between the two cases because there are not only a lot of tests that overlap, but also the differences. So, they were alike in some ways, but each had its own unique aspects. (FG1, S1)*

*You have to think it through logically yourself. For example, when you see a test, you need to ask why it would be necessary if the tumour were in the lung instead of the head and neck. (The what-if questions) pushes you to consider the reasoning behind each decision. (FG1, S2)*

#### Theme 4: The peer dialogue on VP system feedback facilitated concept abstraction

Students described feedback from the VP system as an important trigger for reflecting on their clinical decisions. Discussing this feedback with peers helped them explain their reasoning, recognize mistakes, and better understand why particular diagnostic or treatment choices were appropriate. During these discussions, they explained how they could use the feedback to solve a challenging novel case.

*We looked at the mistakes we made. I really thought the explanations helped, because we could explain things to each other. We could correct ourselves and help each other understand why it was like that… I also think you can apply the feedback from the previous case to the next one. Each time, you understand it better and better. (FG1, S2)*

*For the lung case, that was quite a new case, a different context … but you could use what you learned from the head and neck cases to make decisions. (FG1, S1)*

## Discussion

This study set out to evaluate the effectiveness of the intervention, to determine whether students improved their performance on cases requiring both near and far transfer, and to understand how and why participants experienced the intervention as a means of fostering transfer.

In this intervention, students observed expert role modelling, practiced with varied authentic VP cases, and engaged in “what-if” discussions around cases. From the survey, students responded positively to the intervention. Pre- and post-test results showed improvements in selected aspects of clinical reasoning, particularly in far-transfer tasks. These findings indicate that embedding VPs within a structured sequence of role modelling, varied case practice, and reflective discussion may support knowledge transfer more effectively than using VPs as isolated cases.

The pre- and post-tests showed a mixed pattern. While no significant improvement was observed for overall near-transfer performance, a significant increase was found for the far-transfer task. At the item level, improvements were most evident in tumour-stage reasoning across both transfer tasks and in initial patient impressions for the far-transfer case, whereas no significant gains were observed for test selection or treatment planning. This pattern indicates that the intervention supported specific aspects of clinical reasoning, particularly in the far-transfer context, rather than leading to uniform improvement across all performance components. The absence of improvement in test selection and treatment planning may indicate that these components rely more heavily on procedural or conditional knowledge, which may require more extended practice or clinical exposure than provided in the intervention.

To better understand this pattern, we integrated the quantitative results with the qualitative findings from the group interviews. Taken together, the observed improvements in far transfer and tumour-stage reasoning, and the qualitative reports of recognising recurring reasoning steps, converge to suggest that learners began to move beyond case-specific recall and to identify more generalizable features of clinical reasoning.

The qualitative findings provide insight into how different components of the intervention may have supported learning and transfer. Students responded positively to the role modelling demonstration and practice with varied cases. This pattern is consistent with previous work suggesting that transfer can be supported when learners are scaffolded in complex tasks and when variability of practice is built into the learning environment [4,8]. In the interviews, students described the expert demonstration as giving them a clearer understanding of how to approach later cases, and the repeated case work helped them become more familiar with the overall sequence of reasoning steps. This suggests that role modelling provides students with a cognitive schema and the intervention as a whole provided a structured scaffold that supported students in organizing their clinical reasoning across cases.

The findings also suggest that practice with varied VP cases helped students recognize recurring reasoning steps across patients with different surface features. This supports schema development across contexts. This interpretation aligns with theoretical accounts of transfer that emphasize abstraction and the recognition of structural similarity across contexts [7,11,12,13]. Students’ descriptions of seeing “similar steps” across cases suggest that they were beginning to identify more generalizable features of the reasoning process, rather than simply recalling earlier content. This may help explain why the strongest gains were found in tumour-stage reasoning and in the far-transfer case, both of which required students to extend their reasoning beyond the specific context of the practiced cases.

The findings also offer a more nuanced view of the role of “what-if” discussions. Although survey ratings for these discussions were relatively lower than those for role modelling and varied case practice, the interview data suggest that students experienced them as especially valuable for comparing cases, reflecting on mistakes, and understanding why similar reasoning steps might lead to different decisions in different patient situations. This interpretation aligns with earlier work suggesting that “what-if” reasoning and structured reflection can help learners connect concrete decisions to broader principles and thereby support transfer [19,20,21,22]. Rather than functioning simply as a review activity, the discussions appear to have encouraged students to examine how differences in age, comorbidities, and context could alter diagnostic and treatment reasoning. In this sense, the “what-if” prompts may support schema abstraction and have served as a bridge between case-specific feedback and more generalizable clinical understanding.

Relatedly, the interview findings suggest that peer dialogue around VP feedback played an important role in supporting concept abstraction. Students described how discussing the system feedback with peers helped them explain their choices, identify mistakes, and better understand why particular answers were more appropriate. This finding suggests that feedback may have contributed to transfer not only by correcting performance, but also by creating opportunities for collaborative interpretation of the reasoning process. This interpretation fits with the broader view that VPs may be most educationally useful when embedded within a larger activity structure, rather than treated as stand-alone instructional tools [17]. In the present study, role modelling, varied case practice, feedback, and reflective discussion appeared to work together as an integrated sequence in which each component prepared students for the next.

These findings extend existing transfer literature by suggesting that variability of practice alone may be insufficient to support transfer. Instead, transfer appears to emerge from the coordinated sequencing of modelling, practice, feedback, and structured reflection, which together enable learners to move beyond case-specific reasoning toward more generalizable understanding.

### Strengths, limitations and further research

This study’s mixed-methods design, combining surveys, performance measures, and qualitative data in an authentic course setting, provided a rich and ecologically valid picture of the intervention.

Several limitations must also be acknowledged. First, the small sample size diminishes generalizability and reduces statistical power, which may partly explain the mixed pattern of results. Second, the duration of the intervention (110 minutes) may have constrained the extent to which students could consolidate their learning and may be insufficient for procedural knowledge development, which may explain the lack of improvement in test selection and treatment planning. Third, the quality of the “what-if” discussions likely varied across groups, which may have influenced the extent to which students were able to translate improved reasoning into correct diagnostic and treatment decisions, and which might also explain the more positive findings for the perceived effectiveness of the “what-if” discussions in the interview. In addition, the role modelling and discussion were facilitated by an expert educator with substantial domain and teaching expertise [8], which may limit the transferability of the findings to settings with less experienced instructors. Finally, as the intervention combined role modelling, authentic case practice, and “what-if” discussions, the observed effects reflect the integrated design rather than any individual component, making it difficult to isolate the specific contribution of each element. Moreover, as this study was conducted using VP simulations, the findings rely on the assumption that students’ performances in simulated environments reflect how they would reason and act in real clinical practice. Although VPs aim to approximate authentic scenarios, differences between simulated and real-world contexts may influence learners’ engagement and decision-making, and therefore the extent to which observed performance translates to actual clinical settings remains uncertain.

Future research should test longer or repeated interventions. Studies should also examine how the quality of “what-if” discussions affect learning outcomes, for example by comparing different facilitation approaches or providing more structured discussion guides. Finally, replication studies with larger cohorts, across different educational contexts, and experimental studies in which conditions can be controlled are needed to clarify the unique contribution of each instructional element. Future work could also explore how this instructional approach might be embedded longitudinally in the curriculum and what forms of faculty development are needed to help instructors facilitate role modelling and “what-if” discussions effectively.

## Conclusion

This study suggests that VP-based learning supports knowledge transfer when it is deliberately designed as an integrated instructional sequence combining modelling, variability, and structured reflection. The findings do not suggest uniform improvement across all aspects of performance, but they do indicate that such an approach may support the development of clinical reasoning that enables the application of knowledge across different cases, especially when learners are prompted to articulate and compare the reasoning underlying their decisions. Taken together, the findings suggest that the intervention may support both core reasoning processes and the flexible application of knowledge in new contexts.

## Additional Files

The additional files for this article can be found as follows:

10.5334/pme.2363.s1 Appendix 1. The variation of cases.

10.5334/pme.2363.s2 Appendix 2. Virtual Patient system feedback interface.

10.5334/pme.2363.s3 Appendix 3. What-if questions.

10.5334/pme.2363.s4 Appendix 4. What-If Discussion Guide.

10.5334/pme.2363.s5 Appendix 5. Paper Case D and Case E.

10.5334/pme.2363.s6 Appendix 6. Survey on Student Perceptions of Learning and Knowledge Transfer (N = 21).

10.5334/pme.2363.s7 Appendix 7. Rubric for assessing students’ answers.
