# Supplementary material for: Ensuring Knowledge Transfer: The Critical Role of “What-If” Discussions in Virtual Patient Simulation
Source: Perspect Med Educ. 2026 Aug 5;15(1):633–43. doi: 10.5334/pme.2363 (PMC13450267; doi:10.5334/pme.2363)
Supplement: Supplementary file 1 [file pme-15-1-2363-s1.pdf]

Appendix 1: The variation of cases

	Problem type	Patient Factors	Examinations and lab tests	Disease extension	Treatment Plan
**Course case (Case A)**	Neck cancer	67 years old, female, smoked before, 0-1 unit of alcohol per week. No medical history. 4 kg weight lost in last 3 months. WHO PS 0.	Oropharynx,<br>CT, neck ultrasound, chest CT, Boispy	T3, N2, M0	Chemoradiation
**Practice case (Case B: John Brown Neck VP Case)**	Neck cancer	56 years old, male, No smoking history, 1-2 units of alcohol per day. No medical history.<br>3 kg weight lost in last 3 months.<br>WHO PS 0.	Oral cavity, Palpation of neck lymph nodes, flexible panendoscop, MRI, chest X-ray, neck ultrasound	T2, N1, M0	Chemoradiation
**(Case C: Jack Clark Neck VP Case)**	Neck cancer	68 years old, male, stop smoke 8 years ago, 0 units of alcohol per week. Thoracotomy for mitral valve replacement<br>WHO PS 1.	Oral cavity, Palpation of neck lymph nodes, flexible panendoscop, CT, chest X ray, biopsy	T2, N0, M0	Locoregional radiotherapy
**Practice case Near case (Case D: Joe David Neck Case)**	Neck cancer	75 years old, male, 20 cigarettes per day, 4 units of alcohol per day. 2018 surgery for colon carcinoma (radically treated without recurrences), 2022 coronary artery disease for which CABG, hypertension, and atrial fibrillation.<br>5 kg weight lost in last 3 months.<br>WHO PS 2.	Oral cavity, Palpation of neck lymph nodes, flexible panendoscop, CT, neck ultrasound, chest CT, Fine needle cytology, biopsy.	T2, N3, M0	Surgery (neck dissection) followed by locoregional radiotherapy
**Far case (Case E: Mike Evan Lung Case)**	Lung cancer	78 years old, male, 30 pack years of smoke, and quit smoking 6 years ago. 1-2 units of alcohol per weekend. WHO PS 0	General Visual Inspection, blood pressure & HR, Pulmonary, Abdominal palpation, Chest CT, Chest X-ray, Bronchoscopy, General blood tests, PTFs.	T1, N0, M0	local radiotherapy
