# Supplementary material for: Ensuring Knowledge Transfer: The Critical Role of “What-If” Discussions in Virtual Patient Simulation
Source: Perspect Med Educ. 2026 Aug 5;15(1):633–43. doi: 10.5334/pme.2363 (PMC13450267; doi:10.5334/pme.2363)
Supplement: Supplementary file 2 [file pme-15-1-2363-s2.pdf]

Appendix 2: Virtual Patient system feedback interface

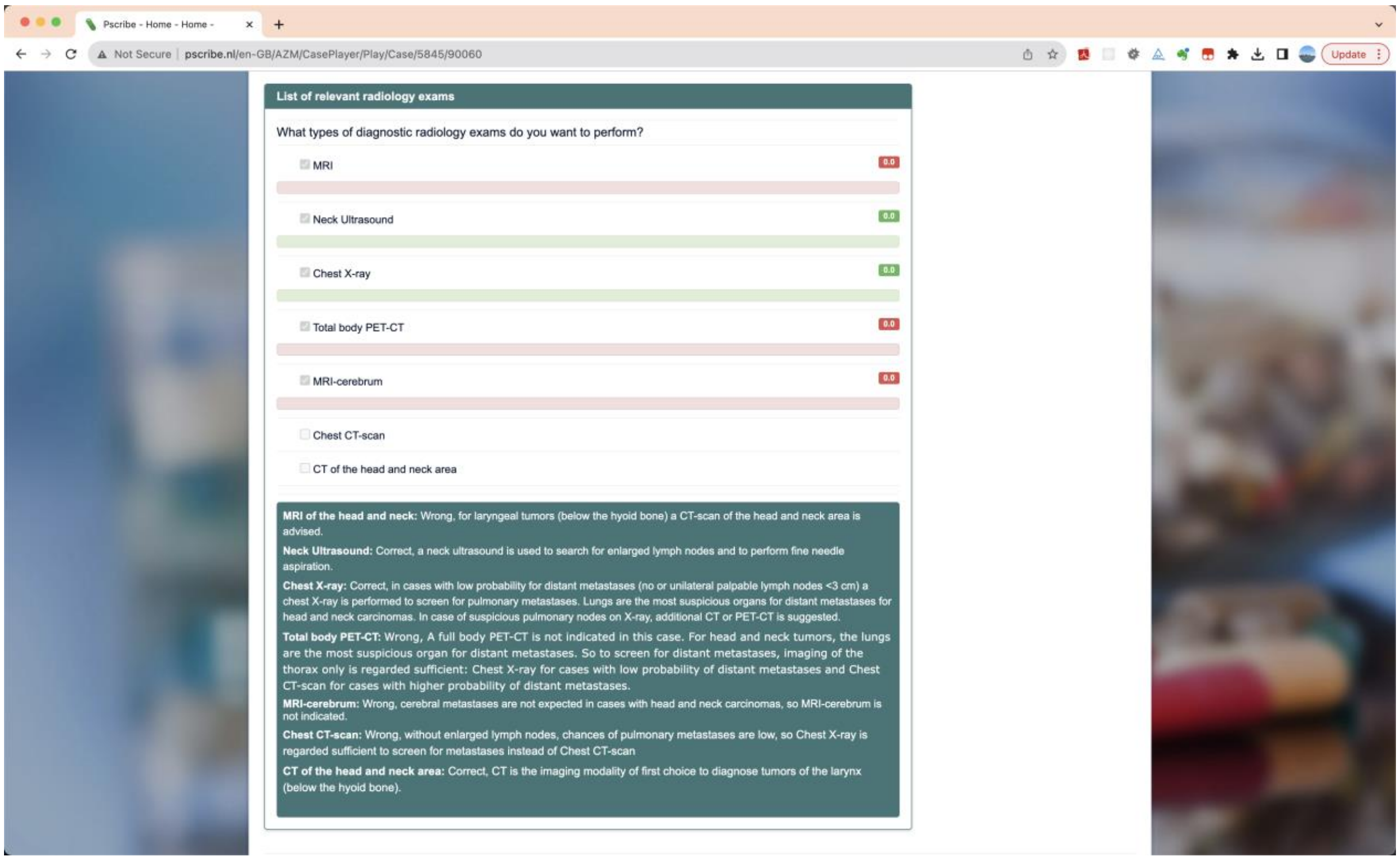

Pscribe - Home - Home -

Not Secure | pscribe.nl/en-GB/AZM/CasePlayer/Play/Case/5845/90058

Update

Case Reflection and Learning Points

Save

Last step. Complete case? Complete

Feedback

- HPV-positive squamous cell carcinoma is most likely to arise in the oropharyngeal region.
- For oropharyngeal carcinomas, MRI is the diagnostic exam of the first choice, as tumors in this region are more visible than on CT.
- For oropharyngeal carcinomas, radiotherapy with or without chemotherapy is the treatment of the first choice, as surgery in this region is expected to have more consequences for organ functions.
- For younger patients without severe comorbidities with stage III-IV disease, chemoradiation is the treatment of first choice.
- If treatment of the first choice is not possible, alternatives should be considered.

Pagelinks

No link found.

General summary

Print feedback | Print (short version)

Made by:	Started on:	Completed on	Printed on:
[redacted]	[redacted] at 00:59	[redacted] 01:32	[redacted] 01:32

Initial situation

List of indications

- Sore throat
- Oncological diseases, other
- Pain associated with cancer

1 - Initial Assessment

1.1 - Physical Examinations

Physical examination

What types of physical examination do you suggest to perform?

Your choices:

- [ ] Inspection of the oral cavity
- [ ] Palpation of neck lymph nodes
