# Supplementary material for: Ensuring Knowledge Transfer: The Critical Role of “What-If” Discussions in Virtual Patient Simulation
Source: Perspect Med Educ. 2026 Aug 5;15(1):633–43. doi: 10.5334/pme.2363 (PMC13450267; doi:10.5334/pme.2363)
Supplement: Supplementary file 3 [file pme-15-1-2363-s3.pdf]

Appendix 3: What-if questions

Aspect	What-if question
**Age and Gender**	What if the patient was an 80-year-old female?
**Co-morbidity Presence**	What if the patient had a history of diabetes or cardiovascular disease?
**Smoking and Alcohol History**	What if the patient was a heavy smoker or a frequent alcohol consumer?
**Advanced Disease State**	What if the patient presented metastatic disease (M1 stage)
**Tumor Location Change**	What if the primary tumor was located in the thyroid gland instead of the original region in your practice?
