# Supplementary material for: Ensuring Knowledge Transfer: The Critical Role of “What-If” Discussions in Virtual Patient Simulation
Source: Perspect Med Educ. 2026 Aug 5;15(1):633–43. doi: 10.5334/pme.2363 (PMC13450267; doi:10.5334/pme.2363)
Supplement: Supplementary file 4 [file pme-15-1-2363-s4.pdf]

Appendix 4: What-If Discussion Guide

First, discuss within your group, and find out what the differences between the VP case you practiced with others'

This guide is designed to help you explore different scenarios based on the virtual patient case. By altering specific patient variables, you will understand how these changes can influence diagnostic and therapeutic approaches.

1. Group Comparison:

First, discuss the virtual patient case within your group. and find out what the differences are between the VP case you practiced with others in your group.

2. Scenario analysis.

Discuss within your group, and think of the following question in each scenario.

- How do the changes in each scenario impact the diagnostic approach and initial workup?

- What are the key considerations for altering the treatment plan in each scenario?

What-if scenarios

a. Age and Gender Variation

What if the patient was an 80-year-old lady?

b. Presence of Comorbidities

What if the patient had a history of diabetes and cardiovascular disease?

c. Smoking and Alcohol History

What if the patient was a heavy smoker and a frequent alcohol consumer?

d. Advanced Disease State

What if the patient presented with metastatic disease (M1 stage)

e. Tumor Location Change

What if the primary tumor was located in the [new location] thyroid gland instead of the [answered location] tonsillar region?
