# Supplementary material for: Ensuring Knowledge Transfer: The Critical Role of “What-If” Discussions in Virtual Patient Simulation
Source: Perspect Med Educ. 2026 Aug 5;15(1):633–43. doi: 10.5334/pme.2363 (PMC13450267; doi:10.5334/pme.2363)
Supplement: Supplementary file 5 [file pme-15-1-2363-s5.pdf]

Appendix 5: Paper Case D and Case E

Joe David Neck Cancer Paper Case D

Personalized Medicine and Care in Cancer Treatment

Introduction

Welcome to the virtual patient case study. In this exercise, you will play the role of a physician evaluating a patient with potential cancer symptoms. Your task is to gather relevant information, perform necessary examinations, and develop a personalized treatment plan for the patient.

How to use it?

On the Initial Page, you will see a patient, Joe, visiting you. Carefully read through Joe's clinical characteristics, anamnesis, and medical history.

Based on the provided information, choose appropriate examinations from the examination cards, you can flip the examination card to see the results of the examination. Please write down the examinations you choose and your reasoning for choosing those cards.

After you think you get enough information, please use the collected data to provide a diagnosis, determine the TNM staging, and conclude the tumor stage.

Create a personalized treatment plan for Joe, including the reasoning behind each decision.

Initial Page: Patient Information:

**Name**	Joe David	**Age**	75 years
**Gender**	Male	**Height**	178 cm
**Weight**	51 kg	**Job**	Retired

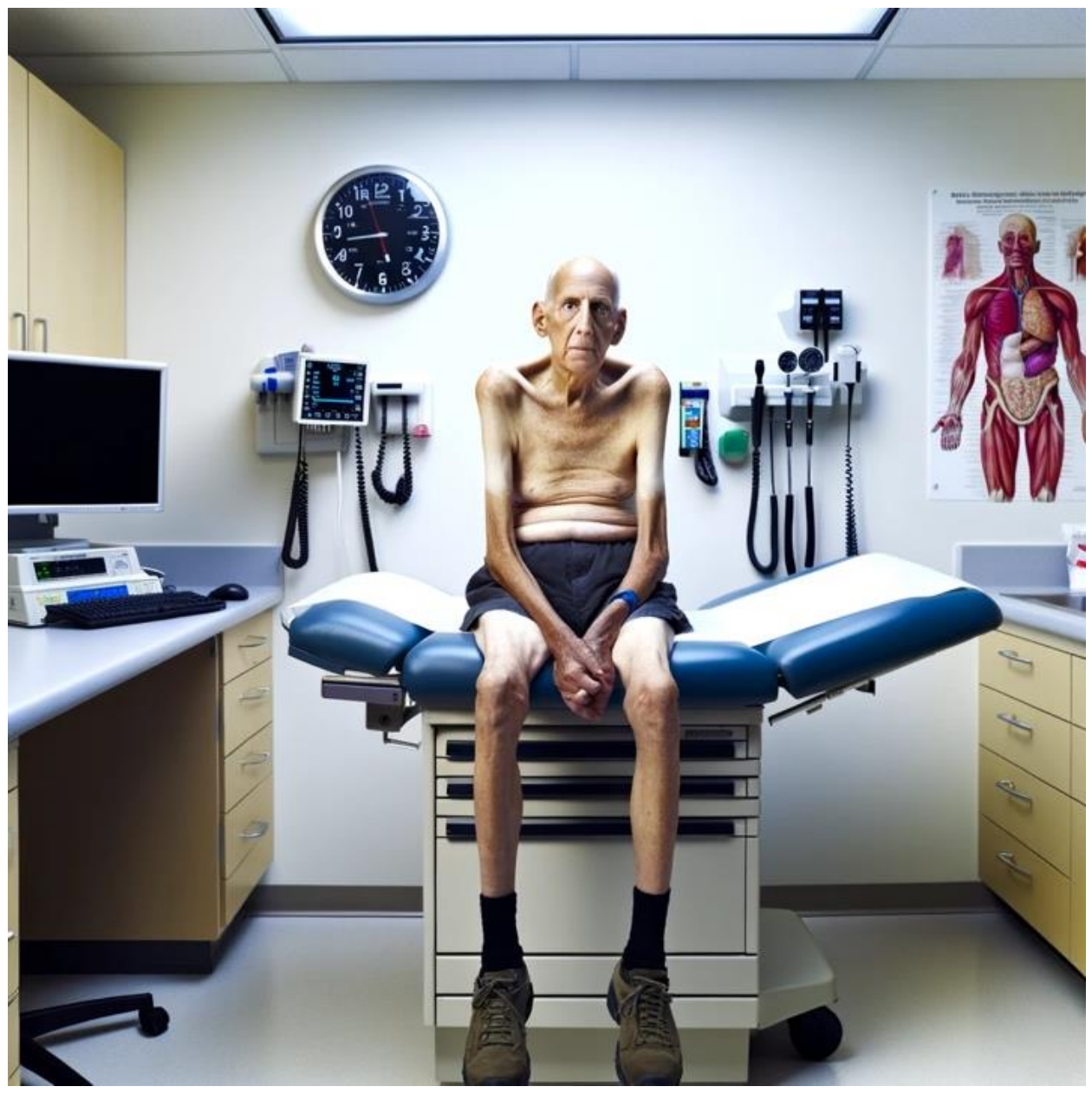

Anamnesis:

- **Physical Performance Status:** Poor, WHO PS 2
- **Complaint:** The patient noticed a growing mass on the left side of his neck for several weeks.
- **Symptoms:**
    - Painful swallowing
    - Disturbed food passage
    - Hoarseness
    - Weight loss (5 kg in the last 3 months)
- **Smoking History:** 20 cigarettes per day since the age of 18 (57 pack years)
- **Alcohol Consumption:** 4 units per day since the age of 18

Medical History:

- **2018:** Surgery for colon carcinoma (radically treated without recurrences)
- **2022:** Coronary artery disease (treated with CABG)
- **Current Conditions:** Hypertension, atrial fibrillation

Reasoning Sheet

Prefer typing instead of writing? Please visit:

Learner's Name:

Scan the QR code:

What is your first impression about the patient?

Pick the examination you need for diagnosing the patient. Flip the cards to see the result of the inspection. Please write down your choices and your reasoning behind it

What is the T, N, M stage?

Please conclude the tumor stage and write down the reasoning behind it

Which treatment should be the treatment of the first choice, according to the patient's characteristics and TNM-stage? Why do you think this treatment plan is suitable of this patient?

Supportive information:

Table 1: WHO performance status

Grade	Performance
0	Able to carry out all normal activity without restrictions.
1	Restricted in physically strenuous activity but ambulatory and able to carry out light work.
2	Ambulatory and capable of all self-care but unable to carry out any work; up and more than 50% of waking hours.
3	Capable of only limited self-care; confined to bed or chair more than 50% of waking hours.
4	Completely disabled; cannot carry on any self-care; totally confined to bed or chair.

Table 2: Simplified protocol for radiologic work up for head and neck cancer

**Evaluation of the primary tumor site**	Tumors above the hyoid bone (i.e. nasopharynx, oral cavity, oropharynx)	MRI of the head and neck area
	Tumors below the hyoid bone (larynx and hypopharynx)	CT of the head and neck area
**Evaluation of regional tumor spread (lymph nodes)**	All tumors	Neck ultrasound
**Screening for distant metastases**	No or unilateral enlarged lymph nodes ≤ 3 cm	Chest X-ray
	Contralateral or bilateral enlarged lymph nodes or nodes > 3 cm	Chest CT-scan

Table 3: Simplified TNM staging for hypopharyngeal carcinoma.

**T1**	Tumor ≤ 2 cm	**N0**	No lymph node metastases	**M0**	No distant metastasis
**T2**	Tumor 2-4 cm	**N1**	One or more ipsilateral lymph node metastasis, max diameter 6 cm.	**M1**	Distant metastasis (e.g. lung, bone)
**T3**	Tumor >4 cm	**N2**	One or more contralateral or bilateral lymph node metastasis, max diameter 6 cm.		
**T4**	Tumor invades surrounding tissue (bone=mandible, deep tongue muscles, skull base or carotid artery)	**N3**	Lymph node metastasis >6 cm.		

Table 4: Simplified treatment protocol for hypopharyngeal carcinoma

T1-T2 and N0	Locoregional radiotherapy
T3-T4 and/or N1-N2, when age <70 and good renal function, minor comorbidity	Locoregional chemoradiation
Any T with N3	-Chemoradiation, if age <70 and good renal function, minor comorbidity.<br>-Otherwise: Upfront neck dissection followed by locoregional radiotherapy

Table 5: Inspection options

A	Inspection of the oral cavity	B	Palpation of neck lymph nodes	C	Pulmonary auscultation
D	Flexible panendoscopy (Nasopharyngolaryngoscopy)	E	MRI of the head and neck	F	Neck Ultrasound
G	Chest X-ray	H	Total body PET-CT	I	MRI-cerebrum
J	Chest CT-scan	K	CT of the head and neck area	L	Biopsy of the left pyriform sinus
M	Fine needle cytology of	N	Whole genome	O	General blood tests

	enlarged lymph nodes		sequencing		including renal function
P	Urine test for hematuria				

Mike Evan Lung Cancer Paper Case E

Personalized Medicine and Care in Cancer Treatment

Introduction

Welcome to the virtual patient case study. In this exercise, you will play the role of a physician evaluating a patient with potential cancer symptoms. Your task is to gather relevant information, perform necessary examinations, and develop a personalized treatment plan for the patient.

How To use it?

On the Initial Page, you will see a patient, Mike Evan, visiting you. Carefully read through Mike's clinical characteristics, anamnesis, and medical history.

Based on the provided information, choose appropriate examinations from the examination cards, you can flip the examination card to see the results of the examination. Please write down the examinations you choose and your reasoning for choosing those cards.

After you think you get enough information, please use the collected data to provide a diagnosis, determine the TNM staging, and conclude the tumor stage.

Create a personalized treatment plan for Mike, including the reasoning behind each decision.

Initial Page: Patient Information:

**Name**	Mike Evan	**Age**	78 years
**Gender**	Male	**Height**	172 cm
**Weight**	60 kg	**Job**	Retired

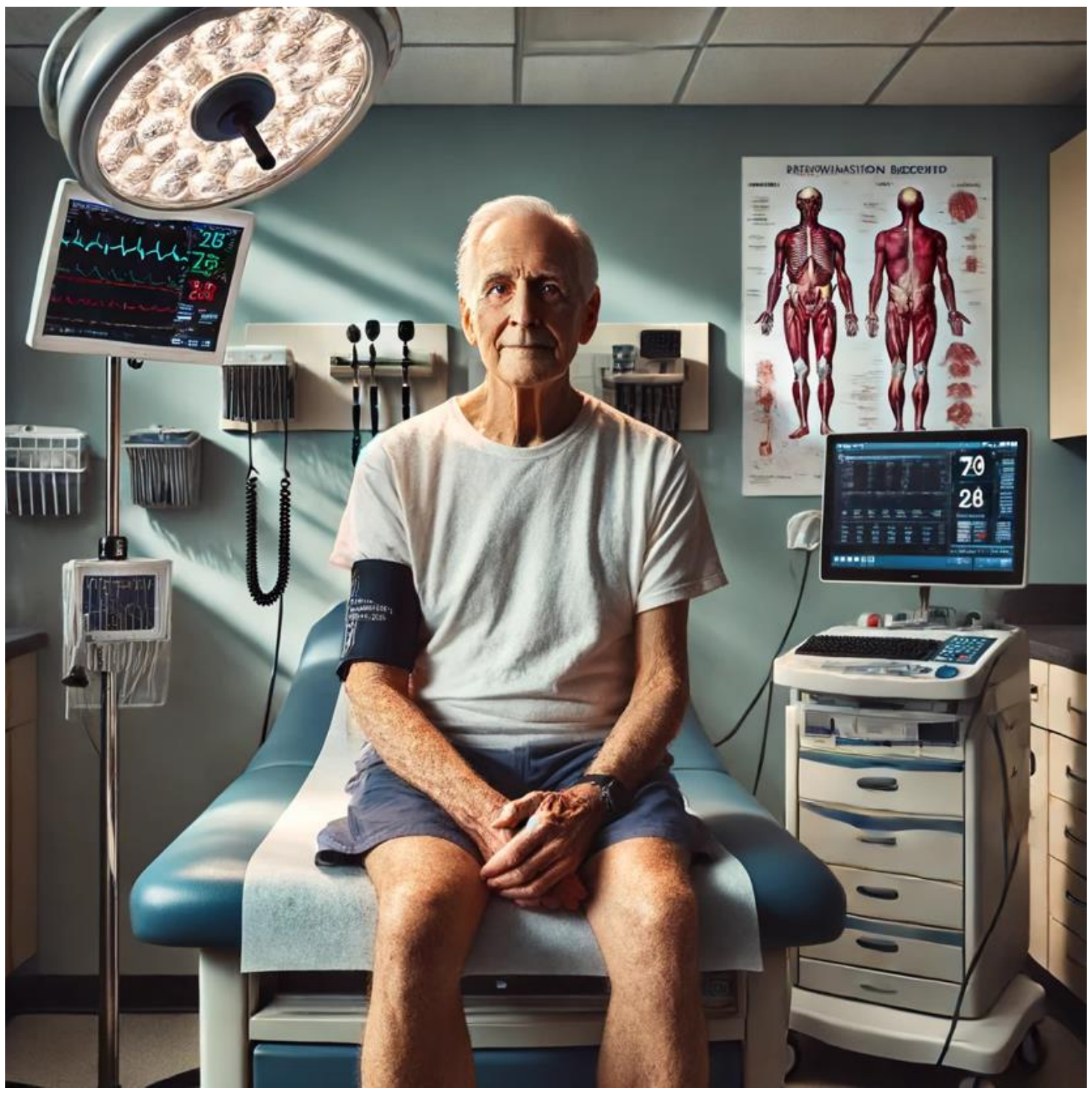

Anamnesis:

- **Physical Performance Status:** He can easily walk, climb a flight of stairs, and walk a couple of kilometers. He lives with his wife and is fully capable of all activities of daily life (WHO PS 0).
- **Complaint:** Fatigue and minor dyspnea after exercise. No cough, no hemoptysis, no weight loss.
- **Symptoms:**
    - No cough
    - No hemoptysis
    - No weight loss
- **Smoking History:** 30 pack years, quit smoking 6 years ago.
- **Alcohol Consumption:** 1-2 units per weekend.

Reasoning Sheet

Prefer typing instead of writing? Please visit:

Learner's Name:

Or Scan the QR code:

What is your first impression about the patient?

Pick the examination you need for diagnosing the patient. Flip the cards to see the result of the inspection. Please write down your choices (A, B, C, …, R) and your reasoning behind it.

What is the T, N, M stage?

Please conclude the tumor stage and write down the reasoning behind it

Which treatment should be the treatment of the first choice, according to the patient's characteristics and TNM-stage? Why do you think this treatment plan is suitable of this patient?

Supportive information:

Table 1: WHO performance status

Grade	Performance
0	Able to carry out all normal activity without restrictions.
1	Restricted in physically strenuous activity but ambulatory and able to carry out light work.
2	Ambulatory and capable of all self-care but unable to carry out any work; up and more than 50% of waking hours.
3	Capable of only limited self-care; confined to bed or chair more than 50% of waking hours.
4	Completely disabled; cannot carry on any self-care; totally confined to bed or chair.

Table 2: TNM-stage for non-small cell lung cancer (TNM 8):

	TNM 8th - Primary tumor characteristics
T_x	Tumor in sputum/bronchial washings but not be assessed in imaging or bronchoscopy
T_0	No evidence of tumor
T_{is}	Carcinoma in situ
T_1	≤ 3 cm surrounded by lung/visceral pleura, not involving main bronchus
$T_{1a(mi)}$	Minimally invasive carcinoma
T_{1a}	≤ 1 cm
T_{1b}	> 1 to ≤ 2 cm
T_{1c}	> 2 to ≤ 3 cm
T_2	> 3 to ≤ 5 cm *or*<br>involvement of main bronchus without carina, regardless of distance from carina or invasion visceral pleural *or*<br>atelectasis or post obstructive pneumonitis extending to hilum
T_{2a}	>3 to ≤4cm
T_{2b}	>4 to ≤5cm
T_3	>5 to ≤7cm in greatest dimension *or*<br>tumor of any size that involves chest wall, pericardium, phrenic nerve *or*<br>satellite nodules in the same lobe
T_4	> 7cm in greatest dimension *or*<br>any tumor with invasion of mediastinum, diaphragm, heart, great vessels, recurrent laryngeal nerve, carina, trachea, oesophagus, spine *or*<br>separate tumor in different lobe of ipsilateral lung
N_1	Ipsilateral peribronchial and/or hilar nodes and intrapulmonary nodes
2	Ipsilateral mediastinal and/or subcarinal nodes
3	Contralateral mediastinal or hilar; ipsilateral/contralateral scalene/ supraclavicular
M_1	Distant metastasis
M_{1a}	Tumor in contralateral lung or pleural/pericardial nodule/malignant effusion
M_{1b}	Single extrathoracic metastasis, including single non-regional lymphnode
M_{1c}	Multiple extrathoracic metastases in one or more organs

Table 3: Inspection options:

A	General Visual Inspection for signs of dyspnea, cyanosis, or anemia	B	Examination of the oral cavity	C	Measurement of blood pressure and heart rate
D	Psychiatric evaluation for mental health	E	Pulmonary and Heart Auscultation	F	Flexible panendoscopy
G	Abdominal palpation	H	MRI-cerebrum	I	Abdominal Ultrasound
J	Chest CT and PET-CT scan	K	Chest X-ray	L	Total body PET-CT
M	Head CT	N	Bronchoscopy with bronchial wash of the left upper lobe	O	General blood tests including renal function
P	Urine test for hematuria	Q	Pulmonary Function Tests (PFTs)	R	Whole genome sequencing
