# Supplementary material for: Ensuring Knowledge Transfer: The Critical Role of “What-If” Discussions in Virtual Patient Simulation
Source: Perspect Med Educ. 2026 Aug 5;15(1):633–43. doi: 10.5334/pme.2363 (PMC13450267; doi:10.5334/pme.2363)
Supplement: Supplementary file 6 [file pme-15-1-2363-s6.pdf]

Appendix 6: Survey on Student Perceptions of Learning and Knowledge Transfer (N=21)

Category	Question	Content	Mean	SD
**Overall Experience**	Q1	My overall experience of the class was positive	4.48	0.51
**Role Modeling (M = 4.44, SD = 0.39, α = 0.50)**	Q2_1	The demonstration by the experienced clinician at the start of the session (Neck Demo Case A) enhanced my understanding of the decision-making process in clinical scenarios.	4.48	0.60
	Q2_2	The demonstration by the experienced clinician at the start of the session (Neck Demo Case A) enhanced my understanding of the reasoning behind the diagnoses.	4.62	0.50
	Q2_3	The demonstration by the experienced clinician at the start of the session (Neck Demo Case A) enhanced my understanding of the underlying principles of the personalized treatment plan.	4.14	0.73
	Q2_4	The demonstration by the experienced clinician at the start of the session (Neck Demo Case A) was useful when I practiced clinical reasoning with Neck VP Case B OR C.	4.52	0.60
**Practice with Various Cases (M = 4.35, SD = 0.61, α = 0.57)**	Q3_1	Engaging myself with Neck VP Case B or C enhanced my understanding of the decision-making process in clinical scenarios.	4.43	0.60
	Q3_2	Engaging myself with Neck VP Case B or C enhanced my understanding of the reasoning behind the diagnoses.	4.43	0.60
	Q3_3	Engaging myself with Neck VP Case B or C enhanced my understanding of the underlying principles of the personalized treatment plan.	4.19	0.51
	Q3_4	The provided Neck VP Case B or C fitted well with my current level of understanding	4.33	0.73
**What-if Discussion (M = 3.76, SD = 0.81, α = 0.90)**	Q4_1	The "what-if" discussions helped me understand the decision-making process in clinical scenarios	3.62	0.80
	Q4_2	The "what-if" discussions helped me understand the reasoning behind the diagnoses.	3.67	0.91
	Q4_3	The "what-if" discussions helped me understand the underlying principles of the personalized treatment plan.	3.76	0.77
	Q4_4	The “what-if” discussion helped me to better understand similarities and differences between the cases.	3.86	0.96
	Q4_5	The “what-if” discussion helped me transfer knowledge from what I learned to similar situations.	3.86	0.73
	Q4_6	The “what-if” discussion helped me transfer knowledge from what I learned to novel situations.	3.81	0.75
	Q4_7	The “what-if” discussion will enable me to apply what I have learned to real clinical practice.	3.76	0.83
**Knowledge Transfer (M = 4.33, SD = 0.70, α = 0.74)**	Q5_1	The entire Virtual Patient session, including the demonstration, practice of virtual case, and what if discussions, helped me transfer knowledge from what I learned to similar situations.	4.48	0.60
	Q5_2	The entire Virtual Patient session, including the demonstration, practice of virtual case, and what if discussions, helped me transfer knowledge from what I learned to novel situations.	4.29	0.72
	Q5_3	The entire Virtual Patient session, including the demonstration, practice of virtual case, and what-if discussions, will enable me to apply what I have learned to real clinical practice	4.24	0.77
