# Supplementary material for: Ensuring Knowledge Transfer: The Critical Role of “What-If” Discussions in Virtual Patient Simulation
Source: Perspect Med Educ. 2026 Aug 5;15(1):633–43. doi: 10.5334/pme.2363 (PMC13450267; doi:10.5334/pme.2363)
Supplement: Supplementary file 7 [file pme-15-1-2363-s7.pdf]

Appendix 7 Rubric for assessing students' answers

Criteria	Weight	Excellent (4)	Good (3)	Satisfactory (2)	Needs Improvement (1)
**Initial Patient Evaluation**	**4**				
Accuracy in Interpreting Patient Info & First Impression (Q: What is the first impression about the patient?)	4	Thoroughly captures all relevant details from patient history, symptoms, and medical background. Provides a comprehensive initial impression of patient's condition.	Captures most relevant details but misses minor aspects. Provides a generally accurate initial impression.	Captures some relevant details from patient history and condition but misses key aspects. (e.g patient's symptoms or medical history).	Misses significant details or misinterprets patient information and fails to describe the patient's condition
**Selection and Justification of Exams**	**8**				
Appropriate Examination Selection (9 correct examinations from 15 options)	4	Students Selects all necessary examinations relevant to patient's symptoms	Correctly selects most necessary examinations. Misses one or two examinations	Makes three or four mistakes in selection of examinations.	Makes more than four mistakes in selection of examinations.
Rationale for Examination Choices (Q: Please write down your choices and your reasoning behind it)	4	Provides clear, logical reasoning for each choice. Demonstrates understanding of the diagnostic process.	The explanation of why choosing a certain examination (e.g. chest X-ray) is almost complete or misses minor points	Reasoning of examination choice is incomplete and lacks support	Reasoning of examination choice is weak or absent.
**Diagnosis and Staging**	**8**				
Correct TNM Staging (Q: What is the T, N, M stage?)	4	All stagings are correct	One staging incorrect	Two stagings incorrect	All incorrect
- Tumor Diagnosis Conclusion and reasoning behind it (Q: Please conclude the tumor stage and write	4	Clearly concludes tumor diagnosis and provides logical reasoning to support the diagnosis	Correctly concludes tumor diagnosis but does not fully explain how the	Reasoning is present but lacks support	Reasoning is wrong or absent.

down the reasoning behind it)			examination results led to this conclusion.		
**Personalized Treatment Plan**	**4**				
- Treatment Plan Development (Q: Which treatment should be the treatment of the first choice)	2	Develops correct personalized treatment plan that aligns with patient's condition and TNM stage.	(If there are three or more parts in the treatment plan) The treatment plan is partially correct but incomprehensive.	(If there are two or more parts in the treatment plan) The treatment plan is partially correct but incomprehensive.	Incorrect/missing
- Rationale for Treatment Choices (Q: Please provide your reasoning behind it.)	2	Provides logical reasoning for each treatment choice.	The explanation as to why the treatment is preferred is unclear based on the patient's specific condition and staging.	Reasoning about treatment plan development is present but lacks support	Illogical/ missing
**Documentation and Presentation**	**0**				
- Clarity and Organization	0	Documentation is clear, well-organized, and easy to follow. All required elements are thoroughly completed with attention to detail.	Documentation is mostly clear and organized. Most required elements are completed.	Documentation is somewhat clear but may be disorganized or difficult to follow.	Documentation is unclear, disorganized, and difficult to follow.
**Total Points**	**24**				
